# Optimized protocol for profiling mucosa-associated microbiota from formalin-fixed paraffin-embedded gut tissues from treatment-naïve pediatric patients with Crohn’s disease

**DOI:** 10.3389/fcimb.2026.1885816

**Published:** 2026-07-14

**Authors:** Noora Al-Ali, Haifa Al-Awadhi, Maya Hassane, Suhail Al-Salam, Farah Al-Marzooq

**Affiliations:** 1 Department of Medical Microbiology and Immunology, College of Medicine and Health Sciences, United Arab Emirates University, Al Ain, United Arab Emirates; 2 Department of Pediatric Gastroenterology, Tawam Hospital, Al Ain, United Arab Emirates; 3 Department of Pathology, College of Medicine and Health Sciences, United Arab Emirates University, Al Ain, United Arab Emirates; 4 Zayed Center for Health Sciences, United Arab Emirates University, Al Ain, United Arab Emirates

**Keywords:** FFPE, gut microbiome, mucosa-associated microbiota, Oxford Nanopore sequencing, pediatric Crohn’s disease

## Abstract

**Background:**

Formalin-fixed, paraffin-embedded (FFPE) tissues are yet underutilized resources for microbiome studies. Data on the mucosa-associated microbiota (MAM) of patients with Crohn’s disease (CD) are scarce, due to several methodological limitations. In this study, we aimed to develop and validate a new optimized amplicon-based workflow to profile MAM from FFPE gut biopsies of pediatric CD patients.

**Methods:**

We examined 68 FFPE samples, including 34 biopsies from treatment-naïve patients with CD and 34 from healthy controls (HC). V3-V7 regions of the 16S rRNA gene were amplified and sequenced on the Oxford Nanopore platform. Two protocols were tested: Protocol 1 (P1), consisting of a single PCR amplification and purification step, and Protocol 2 (P2), including two sequential PCR amplifications with purification after each round. The second amplification and purification steps were introduced to increase sequencing yield and improve microbiota detection.

**Results:**

P2 consistently outperformed P1, yielding significantly higher DNA concentration and purity, reducing human DNA contamination and sustaining pore performance. P2 also generated more microbial reads and recovered a richer, more taxonomically diverse community, including increased detection of species with low abundance. More taxa were enriched in P2 across all levels, enhancing species-level resolution. P2 enabled comprehensive detection of pathogenic genera, such as *Escherichia, Mycobacterium*, and *Klebsiella*, which were significantly enriched in the P2 samples compared to the P1 samples. Alpha diversity analysis showed increased richness and reduced evenness in P2 compared to P1, with a significant difference in beta diversity, while maintaining community structure in both CD and HC.

**Conclusions:**

The optimized workflow with a two-step strategy improved sequencing performance and enhanced microbiota detection in FFPE tissues. This approach enabled successful profiling of MAM, providing a novel method for retrospective characterization of the microbiome from archival tissues and providing a scalable platform for clinical biomarker discovery.

## 1 Introduction

Inflammatory bowel disease (IBD) is a chronic idiopathic relapsing inflammatory disorder of the gastrointestinal tract (GIT). Nearly 3.9 million females and 3.0 million males suffer from IBD worldwide (Alatab et al., 2020). Crohn’s disease (CD) is a chronic IBD marked by recurrent episodes of inflammation, epithelial damage, and aberrant immune responses (Hracs et al., 2025). CD involves mostly the terminal ileum and the large intestine; however, inflammation may involve any segment of the GIT from the mouth to the anus (Alatab et al., 2020). CD is characterized by patchy, transmural lesions that include deep fissuring ulcers, inflammatory infiltrates with granulomas, and lymphoid aggregates (Libertucci et al., 2018). The host immune response in the GIT is interlinked with commensal microorganisms. Mounting evidence indicates that the intestinal microbiota is involved in disease onset and progression, with microbial dysbiosis considered a central factor in CD pathogenesis (Cadwell and Loke, 2025). Over the last two decades, characterization of the gut microbiome has focused more on stool, which may not reflect the composition of the microbiota at lesional sites, where microbial communities most relevant to inflammation are present. Fecal samples, although accessible, capture luminal microbiota and often fail to detect mucosal-adherent microbes that play a direct role in host– and host-commensal interactions at the mucosal surface. They also overlook spatial variation across distinct gut regions, limiting the resolution of microbial profiling (Mottawea et al., 2019; Lam et al., 2021). In contrast, the mucosa-associated microbiota (MAM), which colonizes the gut epithelium and interacts closely with the immune system, is more likely to influence disease activity. Thus, profiling MAM offers a context specific, anatomically resolved view of host-microbe dynamics, particularly at sites of inflammation. Several studies have demonstrated the importance of this approach, emphasizing that MAM differ both taxonomically and functionally from fecal communities (Daniel et al., 2021).

Despite its value, access to mucosal tissue is often limited by ethical and clinical constraints, particularly in pediatric populations, which are generally less well investigated than adults. Endoscopic biopsies are invasive and are typically performed only once during the initial diagnosis. This constraint limits prospective mucosal sampling, especially from treatment-naïve patients. In this context, formalin-fixed paraffin-embedded (FFPE) tissues represent a largely untapped but promising resource for retrospective microbiome research. FFPE samples are routinely collected during diagnostic endoscopy and stored long-term in pathology archives, preserving both histological and molecular features. They offer a unique opportunity to study the mucosal microbiota at the time of diagnosis, before therapeutic intervention, and across diverse anatomical sites (Wyatt et al., 2025). The FFPE preparation process, formalin fixation followed by paraffin embedding, preserves tissue architecture and allows long-term storage of clinical samples. Studies have shown that DNA and RNA can be extracted from FFPE tissues for genetic, epigenetic, and transcriptomic investigations (Lam et al., 2021). In microbiome research, FFPE samples can yield microbial DNA from the mucosal interface, providing insights into bacterial communities within their native tissue context (Pinto-Ribeiro et al., 2020; Lam et al., 2021). However, FFPE samples pose substantial technical challenges for microbiome analysis. Formalin fixation induces cross-linking between nucleic acids and proteins, leading to chemical modifications, fragmentation, and reduced DNA integrity (Singh et al., 2020; Steiert et al., 2023). These alterations complicate PCR amplification, sequencing, and bioinformatic interpretation. Moreover, the low microbial biomass in mucosal samples, combined with overwhelming host DNA content, further limits the recovery of microbial reads and may bias taxonomic profiles. These issues can result in poor reproducibility, reduced diversity detection, and misrepresentation of the microbial community structure (Pinto-Ribeiro et al., 2020; Borgognone et al., 2021).

Recent technological advances have begun to address these limitations. Long-read sequencing platforms such as Oxford Nanopore can tolerate degraded and fragmented DNA, making them suitable for FFPE-derived samples (Kerbs et al., 2025). Additionally, optimized DNA extraction methods and host DNA depletion strategies have improved the recovery of microbial signals. There is a pressing need to develop and optimize reproducible protocols that maximize microbial DNA yield from FFPE tissues, minimize host DNA contamination, and ensure reliable amplification and sequencing.

In this study, we address this gap by developing an optimized, reproducible protocol for microbial profiling from FFPE mucosal biopsies. We applied this workflow to treatment-naïve pediatric CD patients and healthy controls using Oxford Nanopore sequencing via a two-step strategy to improve DNA quality and reduce host contamination. We evaluated the protocols’ performance in terms of DNA yield, sequencing efficiency and quality, microbial diversity, and taxonomic resolution.

## 2 Methods

### 2.1 Ethical approval

FFPE tissue biopsies were obtained from the pathology department of Tawam Hospital, Al Ain, United Arab Emirates. The study protocol was approved by the Department of Health, UAE (reference number DOH/CVDC/2021/1731).

### 2.2 FFPE samples from study participants

This study is retrospective in nature, investigating archived FFPE gut tissues collected from two pediatric groups: treatment-naïve CD patients and healthy controls (HC). A total of 51 FFPE gut biopsy blocks collected between 2013 and 2022 from CD patients were used for protocol testing and optimization (Phase I). Protocol development and validation followed initial optimization (Phase II), whereby two groups were tested: CD and HC (n=34 in each). For the CD group, biopsies were obtained from children aged 6–16 years who were treatment-naïve at the time of sampling. All biopsies were collected during the initial diagnostic endoscopy when patients first presented with GI symptoms. The HC group comprised biopsies from children aged 7–14 years who underwent endoscopy for indications such as a family history of GI disorders or nonspecific abdominal pain. These individuals were neither suspected of having IBD nor diagnosed with any inflammatory condition. Biopsies from both cohorts were obtained from different regions of the GI. For CD patients, only inflamed sites were included; while corresponding non-inflamed regions were sampled from healthy controls to capture a representative mucosal microbiome profile.

All FFPE tissue samples (CD and HC) underwent histopathological evaluation to determine the presence or absence of inflammation. Hematoxylin and eosin (H&E) staining was performed to assess morphological features. All tissue sections were reviewed, and findings were confirmed by a consultant pathologist.

### 2.3 Protocol optimization

In Phase I, 51 FFPE samples were used to test and optimize the protocol, establish baseline performance, and assess the compatibility of standard protocols with FFPE-derived microbial DNA. This included several initial DNA extractions and sequencing trials. Shotgun metagenomic sequencing (whole genome sequencing – WGS) was attempted using the standard protocols provided by commercial kits for DNA extraction [QIAamp DNA FFPE Advanced Kit (Qiagen, Germany)] and library preparation [Native Barcoding Kit 96 V14 (SQK-NBD114.96), Oxford Nanopore Technologies, UK].

In Phase II, the workflow was gradually optimized, including modifications to DNA extraction, PCR conditions, primer selection, and DNA purification steps. The optimized protocol incorporated targeted 16S rRNA amplicon sequencing to ensure accurate microbial detection and to study the microbial makeup using FFPE-derived DNA. These optimization steps aimed at reducing human DNA contamination which is a major challenge when analyzing FFPE samples. These samples are derived from human tissues and therefore contain a high proportion of host DNA, which can obscure microbial DNA detection. To address this issue, we developed an optimized workflow designed to enrich bacterial DNA while reducing host DNA interference. In Protocol 1 (P1), bacterial genomic DNA was selectively amplified using PCR, followed by purification of the PCR products to remove non-target DNA, including a substantial proportion of host DNA. However, because FFPE-derived DNA is often highly fragmented, residual host DNA short fragments may still persist after a single amplification and purification step. Therefore, Protocol 2 (P2) incorporated an additional round of PCR amplification and purification to further enrich bacterial DNA and reduce the contribution of host DNA.

A standard operation procedure (SOP) with the optimized sample preparation steps for P1 and P2 isprovided in the **Supplementary Protocol S1**.

### 2.4 Optimized DNA extraction method

DNA was extracted from FFPE tissue sections using the QIAamp DNA FFPE Advanced Kit (Qiagen, Germany) according to the manufacturer’s recommended protocol, with some modifications. To improve recovery from fragmented DNA, deparaffinization was extended to overnight incubation at 56 °C, and proteinase K digestion was prolonged to 2 hours. The DNA elution time was also extended for 5 minutes to maximize the DNA yield. Following extraction, DNA integrity was assessed by agarose gel electrophoresis. DNA concentration and total yield were evaluated by fluorometric quantification using the Qubit 1X dsDNA High Sensitivity (HS) Assay Kit (Invitrogen, USA), while purity was evaluated by Nanodrop spectrophotometer (Thermo Scientific, USA), reporting A260/280 and A260/230 ratios.

### 2.5 PCR amplification

PCR reactions were prepared using LongAmp Taq 2X Master Mix (New England Biolabs, UK), whichcontains Taq DNA polymerase, dNTPs, MgCl_2_, and optimized buffer components suitable for long-range amplification. Specific primer pairs targeting V3-V7 variable regions of the 16S rRNA gene (amplicon sizes ~300–700 bp) were employed to ensure broad microbial coverage using Multiplex PCR as described previously (Albers et al., 2023). The primer sequences and cycling conditions used are listed in **Supplementary Table 1**. The following PCR cycling conditions were applied after optimization of the temperature and duration of each step: initial denaturation was performed at 94 °C for 1 minute (1 cycle). Denaturation was carried out at 94 °C for 20 seconds (30 cycles). Annealing was conducted at 59 °C for 30 seconds (30 cycles). Extension was executed at 65 °C for 30 seconds (30 cycles). Final extension step at 65 °C for 5 minutes (1 cycle). Positive and negative controls were included in each PCR run. DNA from a healthy pediatric saliva sample was used as a positive control to confirm successful amplification, while a no-template control served as a negative control to monitor contamination. Gel electrophoresis was performed to verify successful PCR amplification and detect potential contamination.

### 2.6 DNA purification

We optimized the DNA purification process to maximize recovery of high-quality DNA from PCR products. We used the MinElute PCR Purification Kit (Qiagen, Germany) following the manufacturer’s instructions. To ensure exceptional DNA purity, the protocol includes an additional 1-minute centrifugation step before elution, which effectively removes residual wash buffer that could contaminate the final product. The elution process utilizes 20 µL of elution buffer (EB), comprising 10 mM Tris-Cl at pH 8.5, which is precisely applied to the center of the QIAquick membrane. A crucial 5-minute incubation period allows the elution buffer to thoroughly penetrate the membrane and efficiently release the bound DNA. The final one-minute centrifugation step yields highly purified DNA in the microcentrifuge tube, ready for subsequent applications.

### 2.7 PCR workflows: Protocol 1 and Protocol 2

We compared two workflows to evaluate the impact of purification and reamplification on microbial DNA recovery. In Protocol 1 (P1), we performed a single round of multiplex PCR followed by purification. In Protocol 2 (P2), we used the purified P1 product as a template for a second round of multiplex PCR, followed by an additional purification step. This dual-processing approach yielded 136 purified PCR products, allowing for a direct comparison between P1 and P2 using identical biological material.

### 2.8 DNA quantification

DNA concentration and purity were measured using a Nanodrop spectrophotometer (Thermo Scientific, USA). DNA concentration was accurately quantified using the Qubit 1X dsDNA High Sensitivity (HS) Assay Kit (Invitrogen, USA) with the Qubit 4 Fluorometer (Thermo Fisher Scientific, USA). This method uses a fluorescent dye specific to double-stranded DNA, providing precise concentration measurements for low-abundance samples. The assay was prepared according to the manufacturer’s instructions.

### 2.9 Library preparation and sequencing

We prepared sequencing libraries using the Native Barcoding Ligation Sequencing gDNA kit 96 V14 (SQK-NBD114.96), provided by Oxford Nanopore Technologies (ONT), UK. In the optimized protocol, DNA was pooled after completing the adapter ligation and clean-up step and before initiating the final preparation for sequencing. The pooling was guided by Qubit results to ensure an equimolar mix of DNA samples. This modification ensures a balanced representation of samples, maximizing efficiency and the sequencing outcome. Sequencing was carried out on the MinION Mk1C device (Oxford Nanopore Technologies, UK) using R10 flow cells, with up to 30 samples loaded per flow cell.

### 2.10 Pore activity analysis and sequencing performance metrics

For the assessment of sequencing run quality, pore activity data were extracted from Oxford Nanopore comprehensive run reports. The number of available pores and elapsed sequencing time (Time-hr) were obtained for each run. Initial and final pore counts were defined as the first and last recorded values of available pores, respectively. Each run was treated as an independent replicate. Total pore activity was calculated for each run as the area under the available pore count curve over time (AUC) using the trapezoidal rule, a standard numerical integration method (Press, 2007), and is expressed in pore·hours. Pore decay was also represented as the percentage of initial pores remaining over time. Differences in initial pore counts and total pore activity between protocols were assessed using one-way ANOVA.

Sequencing output metrics were extracted from ONT run summary reports, including total estimated bases, reads generated, bases passing/failing basecalling, and estimated N50. To assess sequencing efficiency, bases per pore-hour were calculated for each run.

Differences between protocols were assessed using one-way ANOVA. Assumptions of normality (Shapiro–Wilk test) and homogeneity of variance (Levene’s test) were evaluated. When assumptions were met, *post hoc* comparisons were performed using Tukey’s honestly significant difference (HSD) test. When assumptions were violated, the Kruskal–Wallis test was followed by Dunn’s test with a Bonferroni correction. All analyses and visualizations were performed in R version 4.4.0 using the packages dplyr, ggplot2, ggpubr, and dunn.test.

### 2.11 Microbiota bioinformatics analysis

We analyzed sequencing reads using the Kraken taxonomic classification tool (Wood and Salzberg, 2014) and processed operational taxonomic units (OTUs) in the MicrobiomeAnalyst 2.0 platform (McGill University, Canada) (Lu et al., 2023). Sequencing depth and quality were assessed using rarefaction curves and Good’s coverage, calculated as 1 – (F1/N), where F1 represents the number of singleton OTUs and N is the total number of individuals (Chiu, 2023).

We assessed within-sample microbial diversity (alpha diversity) using the observed, Pielou, Chao1, ACE, Shannon, and Simpson indices and then compared the groups tested via P1 vs P2. Between-sample diversity (beta diversity) was evaluated using principal coordinate analysis (PCoA) based on Bray-Curtis and Jaccard distance metrics, and significant differences in community structure were tested using permutational multivariate analysis of variance (PERMANOVA) (Elzayat et al., 2023; Mesto et al., 2026). We analyzed differential taxonomic abundance at the species, genus, and phylum levels using linear discriminant analysis (LDA) with a threshold score of 1. LEfSe (linear discriminant analysis effect size) was applied to identify microbial biomarkers distinguishing the P1 and P2 groups (Elzayat et al., 2023). The Wilcoxon matched-pairs signed-rank test was used to compare P1 and P2 for variables including the *Firmicutes-*to*-Bacteroidetes* (F/B) ratio, % human DNA, and DNA quantity, as determined by Nanodrop and Qubit. The α-diversity indices and the microbiota’s relative abundances were compared using the Mann-Whitney U test or the Kruskal-Wallis test. Statistical significance was established at p ≤0.05 (Mesto et al., 2026). Visualizations were created using R version 4.4.0.

## 3 Results

### 3.1 Phase I: low DNA recovery and FFPE-associated limitations on whole-genome sequencing

**Figure 1:**
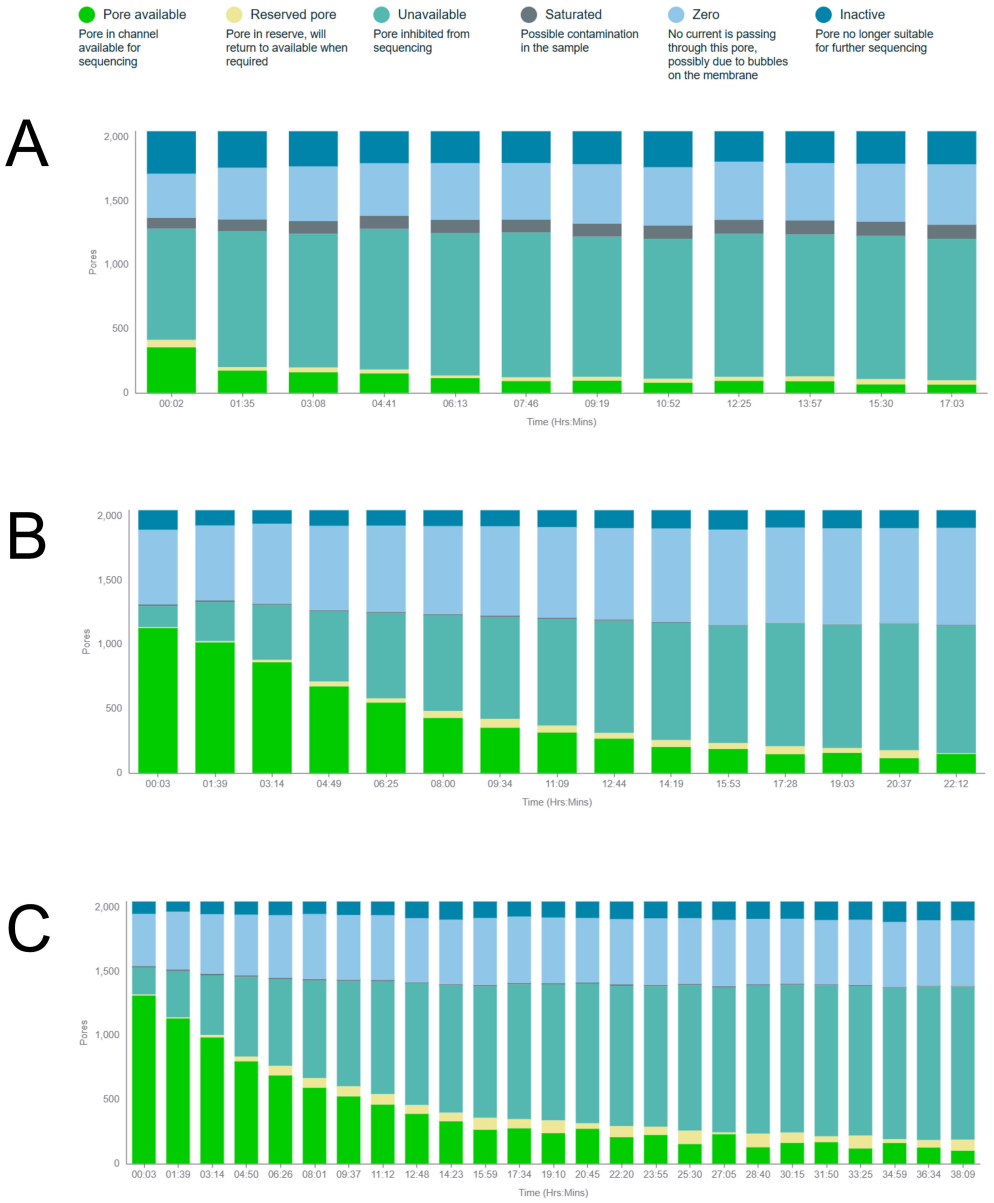
Representative ONT sequencing runs from FFPE samples. The protocol without PCR-whole-genome sequencing **(A)** demonstrates a drop in the number of pores with direct sequencing, while P1 **(B)** and P2 **(C)** showed preserved pore count and longevity.

To achieve comprehensive profiling of the mucosal microbiota, we first attempted direct shotgun metagenomic sequencing. This approach was intended to capture the full range of microbial genes present in the FFPE tissue samples. Upon loading the DNA into Oxford Nanopore flow cells, we observed a rapid and substantial decline in the number of active pores (**Figure 1A**). The MinION Flow Cell typically contains 800–1200 active pores; however, after sample loading, this number dropped immediately to ~400 and continued to decline rapidly. We hypothesized that the blockage of active pores in the flow cells was due to DNA fragmentation and cross-linking, as well as contamination with inhibitors such as paraffin. Shotgun sequencing also failed to generate usable microbial reads due to low microbial biomass and pore clogging. We also assessed DNA integrity by PCR amplification of the 16S rRNA gene and agarose gel electrophoresis. Gel electrophoresis revealed extensive DNA fragmentation, with smearing rather than distinct bands, indicating poor DNA integrity, especially in old samples (> 8 years).

On the grounds of the results, we decided to use FFPE biopsy samples from recent years and adopted a series of optimization steps for our protocol.

### 3.2 Phase II: optimized protocol with multiplex PCR-based workflow and amplicon sequencing

Subsequently, we implemented a series of protocol modifications to address the poor DNA yield and quality observed in Phase I. Optimization steps were applied across multiple stages, including DNA extraction, PCR conditions, primer selection, DNA purification, and library preparation. For DNA extraction, we increased the incubation time in the deparaffinization solution to overnight (original protocol: 1 hr). Furthermore, we increased the incubation time in the lysis step using proteinase K digestion from 1 hr to 2 hrs, and we increased the elution incubation time to 5 minutes.

To ensure reliable microbial detection from FFPE-derived DNA, we adopted an amplicon sequencing approach. Furthermore, we developed a multiplex PCR-based workflow to enhance the sensitivity of microbial profiling. In this strategy, products obtained after the first round of multiplex PCR and purification were designated as Protocol 1 (P1). These P1 amplicons then served as templates for a second round of multiplex PCR and purification, yielding Protocol 2 (P2) products. We validated our workflow using a set of 68 FFPE samples, comprising 34 from treatment-naïve CD patients and 34 from HCs. Each sample was processed through both P1 and P2, resulting in a total of 136 purified PCR products. This dual-processing strategy enabled a direct comparison of P1 and P2 performance using the same biological material, thereby controlling for inter-sample variability and ensuring direct evaluation of the impact of protocol enhancements.

### 3.3 Optimized protocol enhances sequencing performance metrics and flow cell retention

To assess whether improved DNA quality translated into better sequencing outcomes, we monitored the retention of active pore counts on Oxford Nanopore flow cells after library loading. P2 libraries demonstrated superior pore performance, with minimal reduction in active pores post-loading compared to P1, indicating lower flow-cell clogging and better compatibility with sequencing conditions (**Figures 1B, C**).

### 3.4 Purification influences pore stability and cumulative pore activity

**Figure 2:**
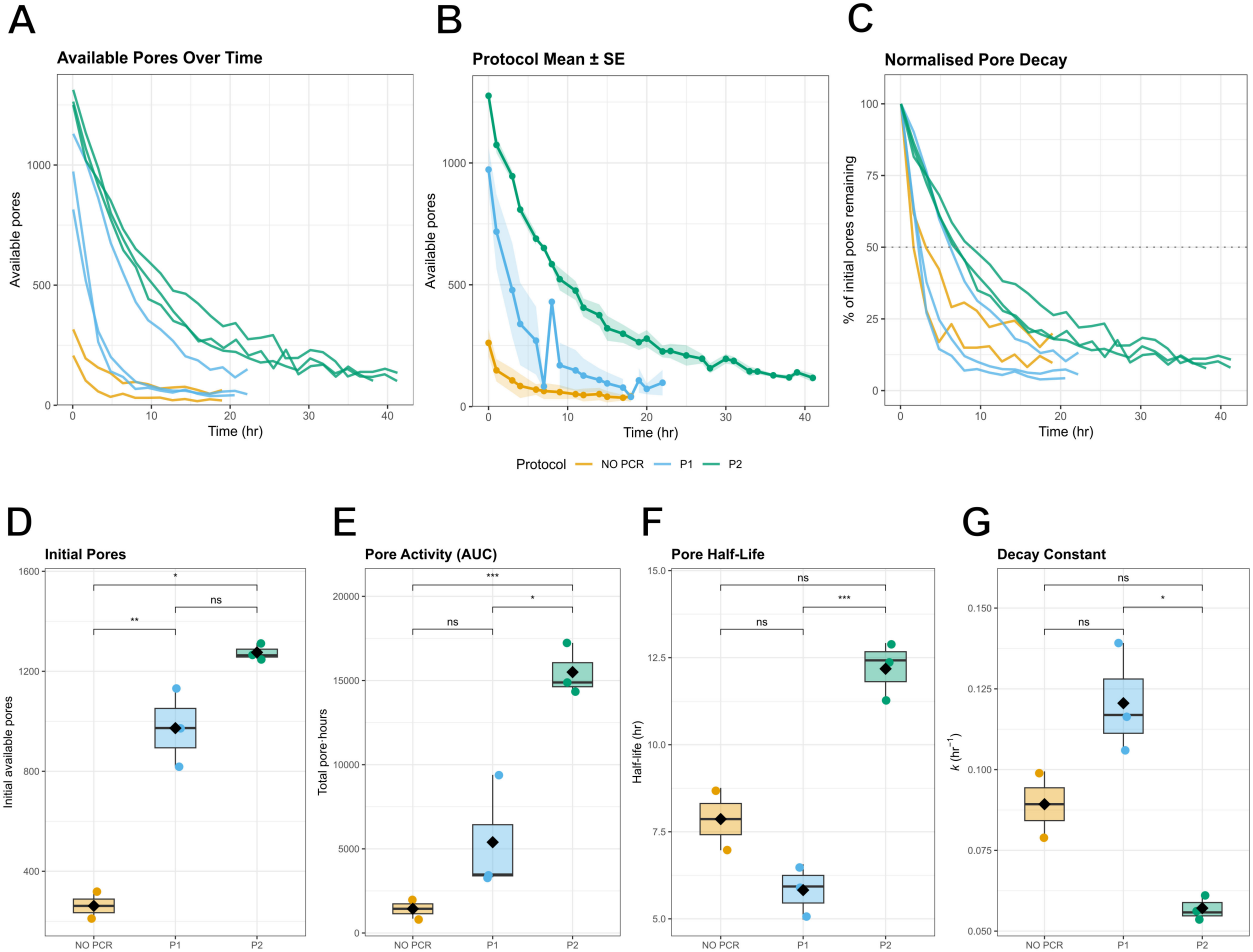
Pore health metrics across library preparation protocols. **(A, B)** Available pores over time for NO PCR (WGS; n=2), P1 (single purification; n=3), and P2 (double purification; n=3) sequencing runs. Individual runs are shown as separate traces, colored by protocol. These panels illustrate differences in initial pore engagement and the temporal decline in active pores during sequencing. **(C)** Normalized pore decay curves expressed as a percentage of the initial available pores. Each trace represents an individual run, enabling direct comparison of decay kinetics independent of starting pore number. A horizontal reference line at 50% indicates the half-life threshold. Boxplots **(D–G)** comparing the initial available pores **(D)**, total pore activity (area under the curve; pore·hours) **(E)**, pore half-life **(F)**, and exponential decay constant (k, hr^−^¹) among protocols **(G)**. Points represent individual sequencing runs; boxes indicate interquartile ranges with medians, and black diamonds denote group means. ns: nonsignificant, *, **, ***: significant difference.

Protocol-dependent differences in pore depletion dynamics were observed, with P2 runs generally exhibiting a slower loss of available pores than P1 and WGS (NO PCR). Available pore counts declined over time (**Figure 2A**), with protocol-level averaging further suggesting improved pore stability in P2 (**Figure 2B**). Normalized decay curves (**Figure 2C**) also confirmed longer pore viability in P2. Initial pore counts (**Figure 2D**) and total pore activity (area under the available pore curve; pore·hours) differed across protocols (**Figure 2E**). One-way ANOVA indicated a significant effect of protocol on cumulative pore activity, and Tukey-adjusted comparisons showed higher total pore activity in P2 relative to at least one other condition. Differences in pore half-life (**Figure 2F**) and decay constant (**Figure 2G**) were directionally consistent with enhanced pore longevity in P2. These metrics collectively assess pore engagement, cumulative pore capacity, and decay kinetics across protocols, as shown in **Figure 2**.

### 3.5 Sequencing yield and read length vary by protocol

**Figure 3:**
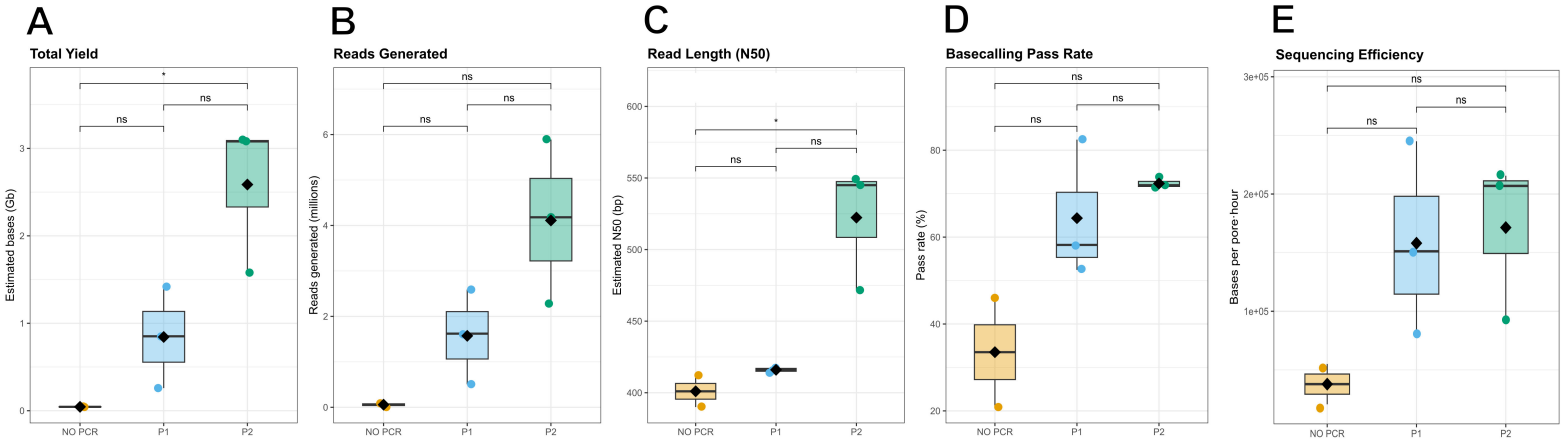
Sequencing output metrics across library preparation protocols. **(A)** Total sequencing yield (Gb), **(B)** reads generated (millions), **(C)** estimated read length (N50, bp), **(D)** basecalling pass rate (%), and **(E)** sequencing efficiency expressed as bases per pore·hour are shown for NO PCR, P1 and P2 protocols. Each point represents an individual sequencing run; boxplots display median and interquartile range, with black diamonds indicating group means. significant difference; * : p ≤0.05.

The total sequencing yield (Gb) differed between protocols (**Figure 3A**). A significant protocol effect was observed, with P2 yielding higher than that of at least one comparator group. Read counts showed similar directional trends (**Figure 3B**). The estimated N50 also differed by protocol (**Figure 3C**). P2 runs exhibited significantly higher N50 values than both NO PCR and P1, whereas P1 did not significantly differ from NO PCR. The basecalling pass rate varied modestly between groups (**Figure 3D**). Yield was strongly associated with cumulative pore activity. Sequencing efficiency, defined as bases generated per pore·hour, differed between protocols (**Figure 3E**), with P2 generally exhibiting higher per-pore productivity. These results demonstrate the impact of library purification on total sequencing output, read counts, read-length distribution, and base-calling quality and confirm the superiority of P2 compared to other protocols.

### 3.6 The optimized protocol successfully amplifies and increases MAM DNA recovery from fragmented FFPE samples

Analytical performance was assessed using multiple quantification and validation methods, including gel electrophoresis to assess amplicon integrity, Qubit fluorometry, and Nanodrop spectrophotometry to assess DNA yield. Gel electrophoresis confirmed successful amplification of multiple 16S rRNA variable regions in both protocols (**Supplementary Figure 1**). Both the P1 and P2 samples displayed multiple bands, consistent with amplification from diverse microbes. Notably, P2 products exhibited more intense, distinct bands than P1, indicating greater amplification efficiency and a higher yield of bacterial DNA. These results highlight the advantage of using shorter amplicons targeting multiple variable regions over the full-length 16S rRNA gene when working with degraded FFPE samples.

**Figure 4:**
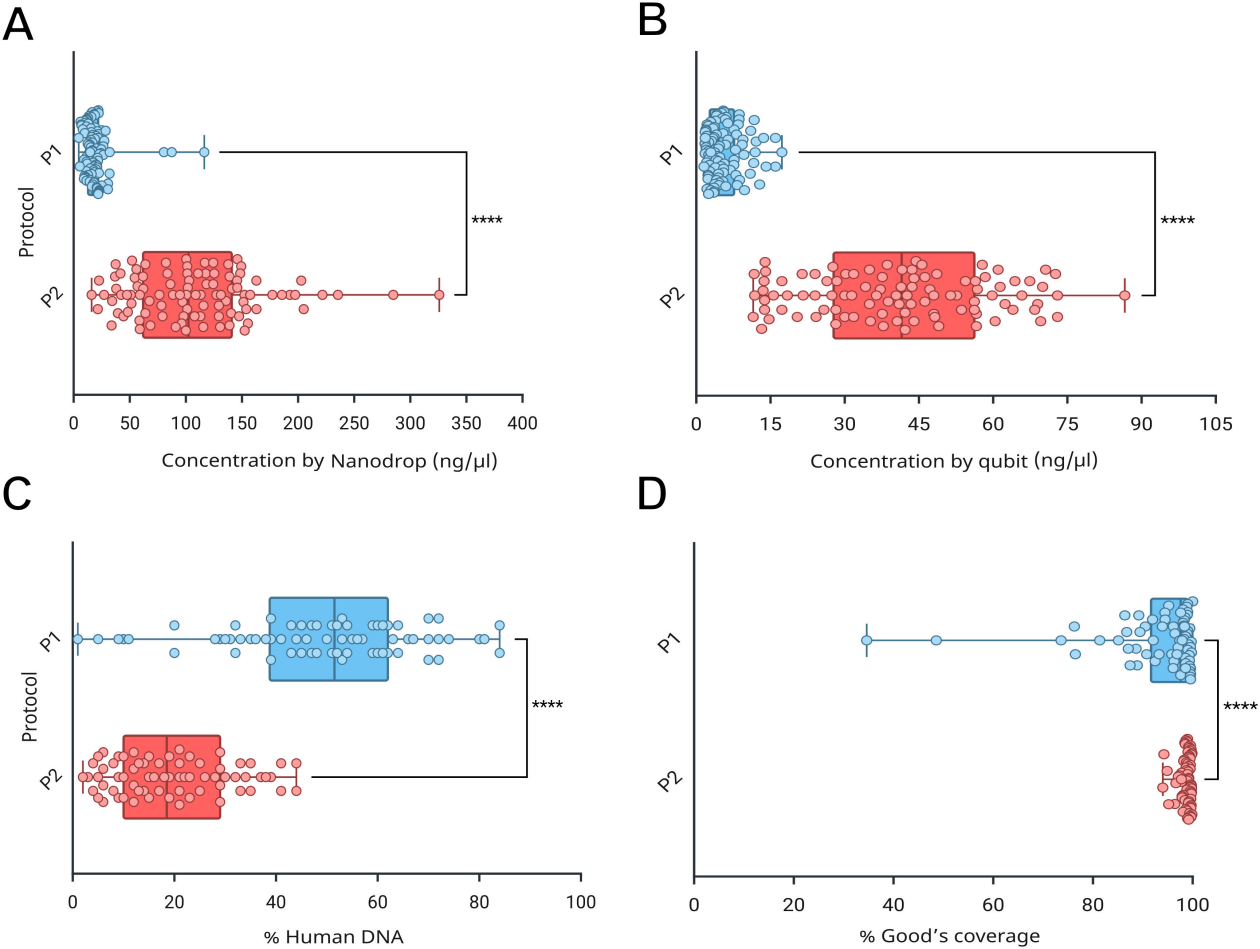
Comparison between P1 and P2 purification protocols. DNA quality and quantity between P1 and P2 measured using nanodrop **(A)** and qubit **(B)**. Percentages of human DNA **(C)** and Good’s coverage index **(D)** are also significantly different. significant difference ; **** : p <0.0001.

The DNA concentration was higher in P2 than in P1 by both Nanodrop spectrophotometry (median = 59.0 vs. 15.4 ng/µL; Wilcoxon matched-pairs signed rank test, p < 0.0001) and Qubit fluorometry (median = 27.4 vs. 4.06 ng/µL; Wilcoxon matched-pairs signed rank test, p < 0.0001), confirming the increased DNA yield achieved by the optimized protocol (**Figures 4A, B**).

### 3.7 Reduced human DNA contamination

Host DNA contamination was estimated as the proportion of human-derived sequencing reads identified. This proportion was then compared between P1 and P2 to evaluate the efficiency of host DNA removal. P2 achieved a mean reduction of 45.96% in host DNA content (median = 63.41%) compared to P1. A Wilcoxon matched-pairs signed-rank test confirmed that this reduction was significant (W = 2242.0, p < 0.001), as shown in **Figure 4C**.

### 3.8 Increased microbial diversity and community coverage

To quantify community coverage, we calculated Good’s coverage values. P1 samples ranged from 34.6% to 99.7%. However, P2 samples consistently achieved values of 94% to 99.9%, indicating near-complete community capture (**Figure 4D**).

**Figure 5:**
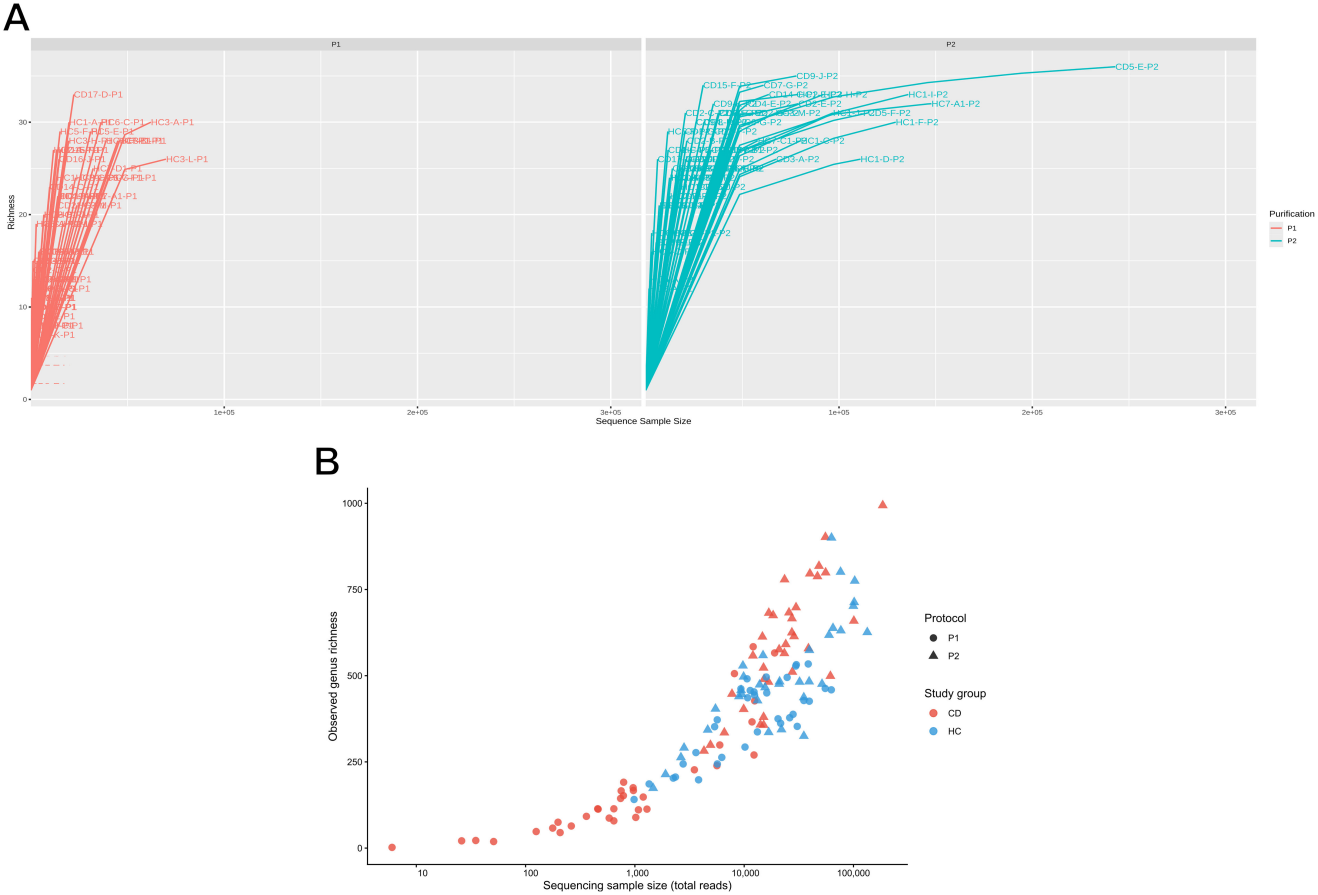
Comparison between P1 and P2 purification protocols. **(A)** Per-sample rarefaction curves showing genus richness as a function of sequencing depth. Each curve represents a single sample; panels correspond to purification protocols (P1 and P2). **(B)** Observed genus richness vs sequencing sample size (total number of reads).

To evaluate the impact of protocol optimization on microbial diversity detection, rarefaction curves were generated for both the P1 and P2 libraries. P2 consistently outperformed P1 across all sequencing depths (**Figure 5A**). The P2 rarefaction curve exhibited a steeper initial slope, indicating greater detection efficiency at lower sequencing depths. Moreover, while the P1 curve plateaued earlier, the P2 curve continued to rise, suggesting improved detection of low-abundance or rare taxa. By looking at the number of reads versus microbial richness (**Figure 5B**), there was a significant increase in the sequencing reads (generated ~43,000 reads/sample) associated with increased richness in P2 compared to P1.

### 3.9 Phylum-level shifts reveal improved detection of low-abundance taxa

**Figure 6:**
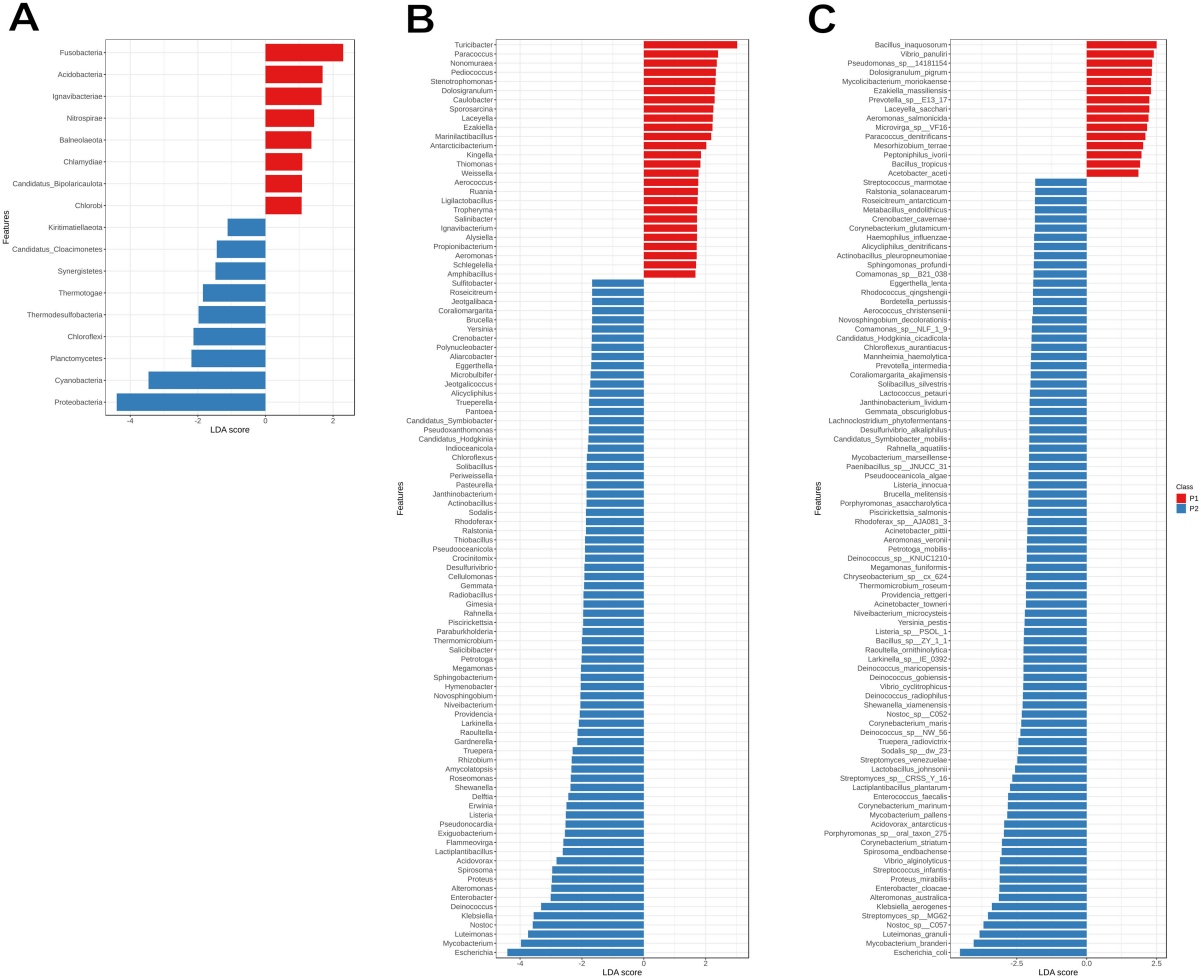
LEfSe analysis. Taxa detected with significantly higher abundance in P2 compared to P1 at the phylum **(A)**, genus **(B)**, and species **(C)** levels are shown.

At the phylum level, P2 enabled the detection of a broader range of bacterial groups, including typically underrepresented phyla in FFPE-derived samples. Specifically, we observed a marked increase in the relative abundance of *Actinobacteria* and *Proteobacteria* in the P2 samples. In contrast, *Bacteroidetes* and *Firmicutes* remained the dominant phyla in both P1 and P2. The apparent dominance of these two phyla in P1 may reflect not only their biological abundance but also the limited sensitivity of single-step amplification, which likely failed to capture low-abundance phyla. This under-detection could lead to biased relative abundance estimates, resulting in overrepresentation of dominant groups. Supporting this interpretation, *Bacteroidetes* and *Firmicutes* were not identified as significantly different between protocols in the LEfSe analysis, suggesting stable representation across both methods. To further assess this relationship, we calculated the *Firmicutes-*to*-Bacteroidetes* (F/B) ratio and compared the P1 and P2 values using the Wilcoxon matched-pairs signed-rank test. The difference was not statistically significant, indicating that P2 did not distort the balance of major phyla but rather enhanced detection sensitivity for lower-abundance groups. LEfSe analysis revealed significant enrichment of *Proteobacteria*, *Cyanobacteria*, and *Planctomycetes* in P2 samples, whereas *Fusobacteria* and *Acidobacteria* were more enriched in P1 (**Figure 6A**).

### 3.10 Genus-level differences highlight enrichment of clinically relevant taxa

At the genus level, P2 enabled more comprehensive detection of pathogenically and biologically important genera. Notably, *Escherichia*, *Mycobacterium*, and *Klebsiella* were significantly enriched in P2 samples compared to P1 samples. These genera include several well-known pathobionts and opportunistic pathogens implicated in intestinal inflammation and dysbiosis. Conversely, *Turicibacter*, *Paracoccus*, and *Helicococcus* were found in higher abundance in P1 (**Figure 6B**).

### 3.11 Species-level profiling demonstrates superior depth and resolution in P2

Species-level LEfSe analysis confirmed that P2 provided significantly enhanced taxonomic resolution. A total of 339 species were found to be significantly enriched in the P2 samples, compared to only 59 species in P1 (**Figure 6C**). This substantial difference underscores the added sensitivity and depth achieved by the dual amplification and purification strategy. Among the most significantly enriched species in P2 were *Streptococcus infantis*, *Enterobacter cloacae*, *Klebsiella aerogenes*, *Luteimonas granuli*, and *Mycobacterium branderi*. Importantly, *Escherichia coli*, a clinically relevant pathobiont frequently associated with gut inflammation, was more prominently detected in P2 samples, further supporting the protocol’s enhanced capacity to recover biologically informative taxa. While some additional taxa detected in P2 had very low relative abundances and may be of limited biological relevance, the ability to detect both common and rare species more reliably demonstrates the robustness of the optimized protocol. These findings suggest that P2 not only increases the sensitivity of microbial profiling but also improves the ability to capture potentially pathogenic species that may be under-represented or missed entirely with single-step protocols.

### 3.12 Diversity of P1 vs P2

**Figure 7:**
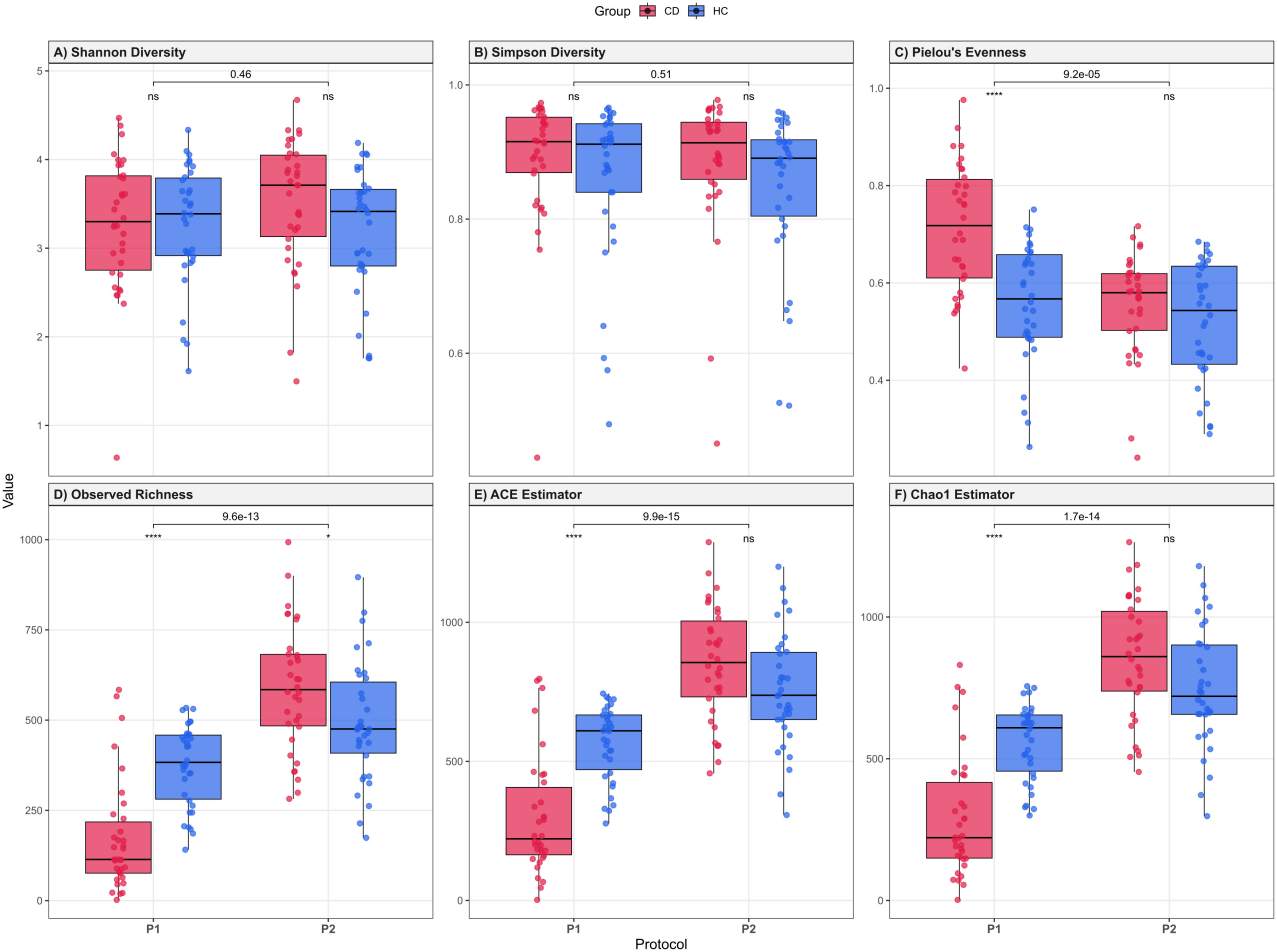
Alpha diversity of P1 vs. P2. Shannon **(A)**, Simpson **(B)**, Pielou**(C)**, Observed **(D)**, ACE **(E)**, and Chao1 **(F)** indices were compared in samples subgrouped into CD and HC. significant difference ; * : p ≤0.05; **** : p <0.0001.

**Figure 8:**
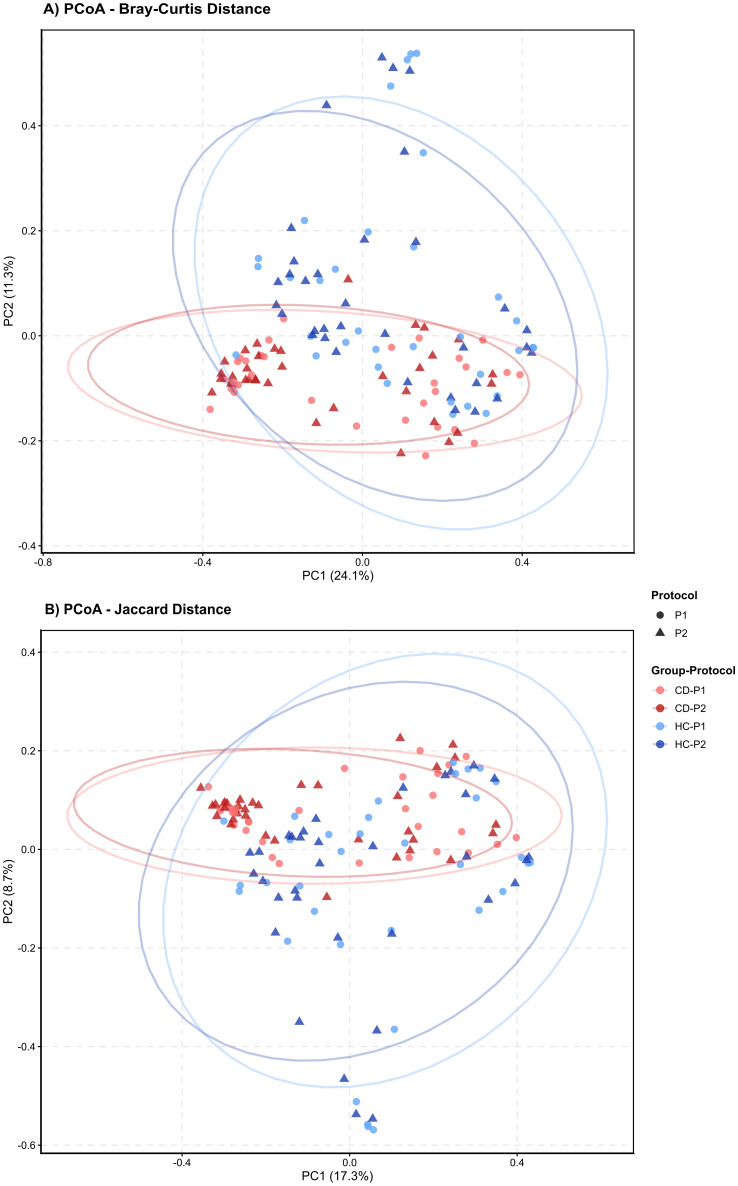
PCoA plots of beta diversity (P1 vs P2). Bray-Curtis **(A)** and Jaccard distance **(B)** metrics were compared in samples subgrouped into CD and HC.

For alpha diversity (**Figure 7**), the observed richness increased substantially in P2 compared to P1. This is reflected in the statistically significant difference (p<0.05), indicating that P2 consistently detects more microbial taxa. P2 showed significantly higher values than P1 (p <0.05) for the Chao1 and ACE diversity indices, whereas the Shannon and Simpson diversity indices were not significantly different between the two protocols (p > 0.05). Interestingly, Pielou’s evenness index was significantly lower in P2 than in P1. Beta diversity was analyzed to compare the microbial community composition between P1 and P2. Both the Bray-Curtis and Jaccard indices showed a statistically significant difference between P1 and P2 (p< 0.05), as shown in **Figure 8**.

Notably, community structure in both CD and HC was maintained, as shown in the beta diversity graphs, which clearly separated the groups in both the P1 and P2 protocols. On the other hand, CD and HC showed significant differences in alpha diversity metrics of either richness or evenness between P1 and P2, while Shannon and Simpson diversity indices (representing both measures) were not significantly different between CD and HC in P1 vs P2.

## 4 Discussion

We established an optimized workflow for profiling the MAM in FFPE gut tissues from treatment-naïve pediatric CD patients. More recently, MAM has been recognized as a key player in health and disease (Darnindro et al., 2025). There is limited information on the taxonomic composition and diversity of MAM in CD, although it may play a role in disease pathophysiology. Additionally, correlation to pathological changes in the gut of CD patients is missing due to the difficulty of obtaining fresh biopsies from diseased individuals. The successful use of Oxford Nanopore Technology (ONT) for profiling the gut microbiome from fresh mucosal samples has prompted researchers to apply it to archival FFPE samples (Darnindro et al., 2025). Furthermore, the discrepancy in biodiversity and taxonomic structure between fecal microbiota and MAM motivated scientists to shift their focus to the mucosal microbiome (Jiao et al., 2022). Our optimized protocol for MAM characterization utilized ONT. Prior to its use in microbiome research, this technology has been used on FFPE tissues for disease characterization. Kerbs et al., 2025 reported the successful use of ONT sequencing as a cost-effective technology for rapid CNS tumor classification from FFPE tissues. Nevertheless, the quality of extracted DNA was shown to affect tumor classification efficiency, with lower-quality samples requiring longer sequencing times and a higher risk of misclassification (Kerbs et al., 2025). In the latter study, three DNA extraction kits were tested, one of which was the QIAamp DNA FFPE tissue kit, which was proven to be effective in yielding DNA of high quality and quantity. In our study, we used a more advanced version of the QIAamp DNA FFPE Advanced Kit, which is recommended for advanced sequencing applications, including microbiome research (Wyatt et al., 2025). The efficiency of the extraction method was reflected in improved DNA quality and quantity after protocol optimization. Research indicates that using silica-based membrane technology can yield high-quality DNA suitable for downstream genetic analysis (Kazmi et al., 2023). Manufacturers’ recommended protocols were modified by other investigators, who extended reagent incubation times, increased sample volumes, and the number of wash steps, leading to increased final nucleic acid recovery and concentration. Improvements in extraction protocols facilitated downstream molecular applications, including the amplification of longer DNA fragments (up to 725 base pairs) from FPPE tissues (Nguyen et al., 2022). Our results aligned with prior reports, as DNA yield improved when incubation steps were extended. Optimal DNA extraction is more critical for microbiome research from FFPE samples, given the lower abundance of microbial populations compared to host biomass (Hockney et al., 2022). By systematically optimizing extraction and amplification steps, we demonstrate that high-resolution, reproducible microbial profiles can be recovered from archived FFPE blocks, thereby opening these archives to microbiome research.

FFPE tissues are known to yield highly fragmented, crosslinked DNA that is often contaminated with paraffin, leading to poor sequencing outcomes (Lam et al., 2021). Our initial extraction attempts confirmed this, producing low concentration and degraded DNA. Shotgun sequencing using a ligation-based workflow without prior amplification also failed to generate usable microbial reads due to low microbial biomass and pore clogging. Attempts to apply shotgun metagenomics in this context are particularly challenging in FFPE tissues because of excessive host DNA contamination and insufficient microbial content (Han et al., 2020). These results align with broader literature emphasizing the need to standardize pre-analytical variables and manage formalin-induced DNA damage to ensure the accuracy of next-generation sequencing from FFPE-derived material (Robbe et al., 2018). The use of shorter amplicons and multiple target regions in degraded samples is recommended; thus, we implemented a multiplex PCR strategy targeting the V3-V7 variable regions of the 16S rRNA gene. This approach is supported by interlaboratory studies showing that 16S rRNA sequencing provides more consistent and reproducible microbial profiles than shotgun metagenomics, particularly in low-biomass or compromised clinical samples (Han et al., 2020). Notably, amplicon-based sequencing using ONT from FFPE specimens was previously described by Albers et al. (2023), who successfully sequenced 32 tissues from patients with bacterial CNS infections, with results matched to microbiological culture results. We adopted the same strategy using the same PCR primers described; however, the central innovation of our approach was the introduction of dual amplification-purification (Protocol 2, P2). A second round of amplification and purification further enhanced signal quality. Compared with the single-amplification strategy (P1), P2 consistently yielded higher DNA quantity and quality, reduced host DNA carry-over, and improved pore stability during nanopore sequencing. Increased purification steps were associated with improved pore engagement, extended pore half-life, and greater cumulative pore activity. These improvements translated into significantly higher total sequencing yield and increased read length (N50), particularly in the double-purification condition. The strong relationship between total pore-hours and yield suggests that cumulative pore stability is a primary driver of sequencing output. Notably, sequencing efficiency was also enhanced under P2, indicating that purification may improve both pore longevity and per-pore productivity. This supports a mechanistic link between library purity and nanopore performance. We validated P1 versus P2 using a matched set of 68 FFPE samples (34 CD and 34 HC), each processed through both protocols. This design allowed for a direct comparison using identical biological material. Several key metrics were used to evaluate the reproducibility, efficiency, and resolution of the protocol. First, we observed a notable reduction in host (human) DNA contamination in P2 compared to P1. This is especially important in FFPE-based microbiome studies, where host genomic fragments often overshadow microbial DNA (Cruz-Flores et al., 2022). These technical gains were evident across multiple validation metrics: Qubit and Nanodrop measurements confirmed higher DNA yield in P2; gel electrophoresis revealed stronger, clearer amplicon bands, indicating more efficient recovery of microbial DNA; and sequencing runs showed improved pore retention and longevity, consistent with reduced carryover of FFPE-derived inhibitors. Importantly, these improvements did not compromise DNA yield, indicating that the additional purification step efficiently removed contaminants without sacrificing microbial templates.

The benefits of P2 extended to ecological measures of microbial representation. Rarefaction curves revealed broader and deeper community coverage than P1, with greater sensitivity to low-abundance taxa. Good’s coverage values approached 100% in P2, underscoring the enhanced completeness of microbial recovery, while the early plateau in P1 suggests limited detection beyond dominant community members. One potential explanation for this discrepancy is early saturation and blockage of nanopore channels in P1, likely due to residual impurities. Building on improvements in sequencing performance and diversity metrics, we examined how the optimized protocol influenced the taxonomic composition of microbial communities at the phylum, genus, and species levels.

At the phylum level, P2 showed an increased abundance of *Actinobacteria* and *Proteobacteria*, which are typically present at lower levels and often underdetected in FFPE-derived samples. *Bacteroidetes* and *Firmicutes* were the most dominant in P1 due to their high abundance in the gut (Derrien et al., 2019) and thus were not missed. However, this apparent dominance may also be attributed to the inefficient detection of multiple other microbial groups following a single purification step. Missed microbial communities in P1 likely affected relative abundances, leading to a false overrepresentation of dominant phyla, particularly those typically abundant in the GIT. Major phyla, including *Bacteroidetes* and *Firmicutes*, did not appear in the LEfSe analysis, indicating that they were not significantly different between P1 and P2. *Bacteroidetes* and *Firmicutes* remained the dominant phyla in both P1 and P2, consistent with their well-established abundance in the gut microbiome (Derrien et al., 2019). F/B ratio showed no statistically significant difference between the two protocols, suggesting that the additional amplification and purification steps in P2 enhanced the detection sensitivity for less abundant taxa without introducing bias in the representation of dominant phyla.

At the genus level, P2 detected several pathogenically important genera, such as *Escherichia, Mycobacterium*, and *Klebsiella*, which were significantly enriched compared to P1. In contrast, genera such as *Turicibacter, Paracoccus*, and *Helicococcus* were more abundant in P1, potentially reflecting differential sensitivity to purification steps. These observations are consistent with the literature, which reports that differences in amplification strategies can shape microbial profiles (Sinha et al., 2015).

At the species level, LEfSe analysis further demonstrated the superior sensitivity of P2. A total of 339 species were enriched in the P2 samples, compared to only 59 species in P1. This enhancement reflects the improved depth and resolution achieved through additional amplification and purification. Among the most significantly enriched in P2 were *Streptococcus infantis, Enterobacter cloacae, Klebsiella aerogenes, Luteimonas granuli*, and *Mycobacterium branderi.* Importantly, *Escherichia coli*, a clinically relevant gut pathogen, was more prominently detected in P2 samples. While some additional species had very low relative abundance and may be of limited biological relevance, detecting these key taxa highlights the P2 advantage in capturing more informative and potentially pathogenic species that may be missed with single-step protocols. The marked increase in species-level resolution underscores the utility of a dual amplification approach for degraded, low-biomass material.

The methodological advances reported here also carry biological relevance. By minimizing host DNA interference and improving microbial signal recovery, P2 increased the effective microbial sequencing depth and enhanced the detection of low-abundance taxa (Willis, 2019). This is supported by significantly higher observed richness, Chao1, and ACE indices in P2 than in P1, indicating improved recovery of rare microbial members. The additional amplification and purification steps likely contributed to increased library complexity by enriching microbial templates and reducing carryover of inhibitors and nontarget DNA (Jones et al., 2015; D’Amore et al., 2016), resulting in cleaner libraries with broader taxonomic representation. Despite the increase in richness, the Shannon and Simpson indices did not differ significantly between P1 and P2, suggesting that the overall diversity structure remained stable. This finding is consistent with the significantly lower Pielou’s evenness observed in P2, indicating that newly detected taxa were predominantly low-abundance members that increased richness without substantially altering the proportional dominance of the core taxa. Thus, while alpha diversity metrics sensitive to richness were influenced by protocol modifications, the dominant community structure remained preserved (Silverman et al., 2021).

Importantly, beta diversity analyses demonstrated consistent separation between CD and HC across both protocols. Significant differences in both Bray-Curtis (abundance-weighted) and Jaccard (presence/absence-based) distances between P1 and P2 confirm that protocol modifications affect both taxonomic detection and relative abundance estimates. Nevertheless, the preservation of disease-associated clustering across workflows indicates that core microbial signatures distinguishing CD from HC are robust to methodological variation. More broadly, the ability to reliably profile treatment-naïve CD patients without therapeutic confounders underscores the value of FFPE archives for investigating disease-specific microbial signatures independently of treatment effects.

Our findings expand on recent studies that applied FFPE tissues to microbiota research. For example, Wyatt et al. (2025) demonstrated the feasibility of FFPE-based profiling using Illumina MiSeq sequencing of the V4 region, achieving modest read depth (~6,600 reads/sample) and 89%-98% coverage. In contrast, our multiplex design targeting the V3-V7 regions, combined with nanopore sequencing, generated ~43,000 reads/sample and nearly complete coverage (up to 99.9%) in P2. Crucially, whereas Wyatt et al. (2025) restricted analysis to freshly archived FFPE tissues (<1 month old), our protocol successfully recovered complex microbial profiles from blocks stored for up to eight years. In a study by Albers et al. (2023), ONT identified bacteria in 12-year-old FFPE tissues recovered from the brain. Given that the latter study used samples from a sterile site during episodes of bacterial infection, the microbial communities are less complex than those in the gut (Arabi et al., 2023). These differences underscore the robustness of the optimized workflow for gut MAM characterization and highlight the importance of sequencing strategies and preanalytical processing in shaping microbiota recovery from gut FFPE samples.

Some of the limitations of this study are related to its retrospective nature, which limited the availability of matched fresh tissues or fecal samples for direct comparison and validation of the microbial profiles obtained. Furthermore, FFPE tissues often contain fragmented and chemically modified DNA due to fixation and long-term storage with residual host DNA that could influence microbial detection. Despite these limitations, the proposed workflow offers significant utility for microbiome research using archived FFPE specimens, which represent a valuable and often underutilized resource in clinical biobanks. The method is particularly suitable when fresh samples are unavailable. The workflow may facilitate microbiome characterization in historical cohorts and support the identification of potential microbial biomarkers associated with disease. However, results should be interpreted with consideration of sample quality, DNA fragmentation, and the potential effects of FFPE processing on microbial DNA recovery.

## 5 Conclusions

Together, our findings establish a robust and reproducible workflow for profiling the mucosa-associated microbiome from FFPE gut tissues. By integrating dual amplification and enhanced purification, the optimized protocol improves pore stability, sequencing depth, taxonomic resolution, and reproducibility compared with single-step approaches. Importantly, it enables reliable recovery of microbial communities from archival material, thereby unlocking FFPE repositories for retrospective microbiome research.

This workflow expands opportunities to study treatment-naïve pediatric CD and other conditions where fresh tissue is limited. By facilitating spatial and taxonomic characterization of archived mucosal samples, the protocol provides a practical platform for investigating region-specific gut dysbiosis, inflammation-associated microbial signatures, and host-microbe interactions in translational and rare-disease settings. Future studies applying this workflow across diverse diseases will further expand the insights obtainable from preserved clinical material.

## Data Availability

The datasets presented in this study can be found in online repositories. The names of the repository/repositories and accession number(s) can be found in the article/**Supplementary Material**.

## Supplementary material

Supplementary Table 1 Multiplex PCR primers for V3-V7 regions of the 16s rRNA gene.

Supplementary Figure 1 Gel electrophoresis comparison of P1 and P2.

Supplementary Protocol S1 Standard operating procedure (SOP) describing the methods used for DNA extraction from FFPE gut-tissue sections, preparing and sequencing the amplicon library on the Oxford Nanopore MinION platform.
