# Supplementary material for: Optimized protocol for profiling mucosa-associated microbiota from formalin-fixed paraffin-embedded gut tissues from treatment-naïve pediatric patients with Crohn’s disease
Source: Front Cell Infect Microbiol. 2026 Jul 14;16:1885816. doi: 10.3389/fcimb.2026.1885816 (PMC13407190; doi:10.3389/fcimb.2026.1885816)
Supplement: Supplementary Table 1 — Multiplex PCR primers for V3-V7 regions of the 16s rRNA gene. [file Table1.docx]

**Table S1. Multiplex PCR primers for V3-V7 regions of the 16s rRNA gene.** These primers were described previously by Albers et al., 2023.

V3-V4: F: 5’-CCTACGGGNGGCAGCAG-3’, R: 5’-GGACTACHVGGGTATCTAATCC-3’

V4: F: 5’-GTGCCAGCMGCCGCGGTAA-3’, R: 5’-GGACTACHVGGGTWTCTAAT-3’

V4-V5: F: 5’-GTGCCAGCMGCCGCGGTAA-3’, R: 5’-CCGTCAATTCCTTTGAGTTT-3’

V5-V7: F: 5’-AACMGGATTAGATACCCKG-3’, R: 5’-ACGTCATCCCCACCTTCC-3’


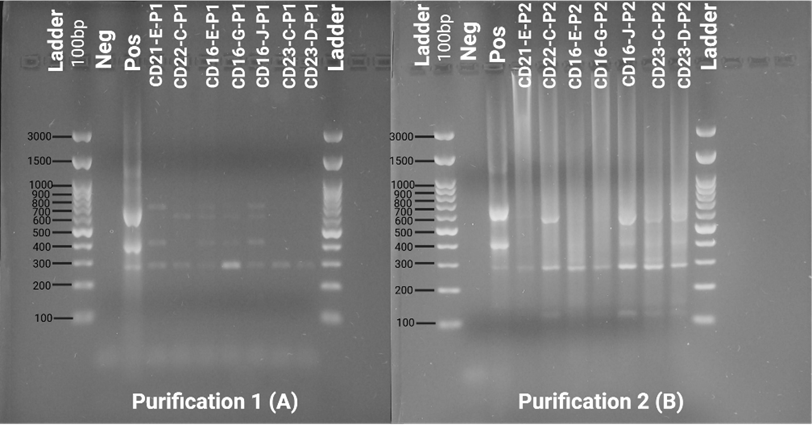


**Figure S1. Gel electrophoresis comparison of P1 and P2.**

**References:**

Albers, A., Spille, D.C., Suero‐Molina, E., Schaumburg, F., Stummer, W., Paulus, W., Thomas, C., 2023. Rapid bacterial identification from formalin‐fixed paraffin‐embedded neuropathology specimens using 16S rDNA nanopore sequencing. Neuropathology Appl Neurobio 49, e12871. https://doi.org/10.1111/nan.12871
