# Supplementary material for: Optimized protocol for profiling mucosa-associated microbiota from formalin-fixed paraffin-embedded gut tissues from treatment-naïve pediatric patients with Crohn’s disease
Source: Front Cell Infect Microbiol. 2026 Jul 14;16:1885816. doi: 10.3389/fcimb.2026.1885816 (PMC13407190; doi:10.3389/fcimb.2026.1885816)
Supplement: Supplementary Protocol S1 — Standard operating procedure (SOP) describing the methods used for DNA extraction from FFPE gut-tissue sections, preparing and sequencing the amplicon library on the Oxford Nanopore MinION platform. [file Table2.docx]

**Optimized protocol for profiling mucosa-associated microbiota from formalin-fixed paraffin-embedded (FFPE) gut tissues from treatment-naïve pediatric patients with Crohn’s disease**

**STANDARD OPERATING PROCEDURE (SOP)**

1. **Purpose:**

This SOP describes the standardized procedure for extracting microbial DNA from FFPE gut-tissue sections, amplifying multiple hypervariable regions of the bacterial 16S rRNA gene, and preparing and sequencing the amplicon library on the Oxford Nanopore MinION platform. The procedure is intended to generate reproducible mucosa-associated microbiota profiles from archival tissues.

1. **Equipment and consumables**

- Microtome and sterile blades for sectioning FFPE blocks
- Thermomixer / heating block (capable of 37 °C, 56 °C, 65 °C and 90 °C)
- Thermal cycler (PCR)
- Benchtop microcentrifuge (capable of ≥15 000 × g)
- Magnetic separation rack for 1.5 mL tubes
- Hula mixer / rotator mixer
- Qubit fluorometer and assay reagents
- Gel electrophoresis apparatus and reagents
- Oxford Nanopore MinION Mk1C sequencer
- Calibrated micropipettes (P10–P1000) and filtered tips
- Sterile, nuclease-free 1.5 mL DNA LoBind tubes and 0.2 mL PCR tubes

1. **Key reagents and kits**

| **Reagent / kit** | **Catalogue number** |
| --- | --- |
| QIAamp DNA FFPE Advanced Kit (Qiagen, Germany) | 56604 |
| LongAmp Taq 2X Master Mix (New England Biolabs, UK) | M0287S |
| PCR primers (Macrogen, Korea) | DPS-025 |
| MinElute PCR Purification Kit (Qiagen, Germany) | 28004 |
| Native Barcoding Ligation Sequencing gDNA kit 96 V14 (ONT, UK) | SQK-NBD114.96 |
| R10.4.1 flow cell (ONT, UK) | FLO-MIN114 |
| Qubit 1X dsDNA High Sensitivity (HS) Assay Kit (Invitrogen, USA) | Q33231 |

1. **Procedures**
   1. **DNA Extraction**

DNA was extracted using QIAamp DNA FFPE Advanced Kit (Qiagen, Germany), as follows:

**Day 1: Deparaffinization**

1. Cut 5–10 µm FFPE tissue sections and transfer them into sterile 1.5 mL microcentrifuge tubes.
2. Add 300 µL Deparaffinization Solution to each sample.
3. Vortex for 10 seconds and briefly centrifuge.
4. Incubate overnight at 56°C.

## **Day 2: Tissue Lysis and Extraction**

##

## **Tissue Lysis**

1. Prepare a master mix containing:
   - 25 µL Buffer FTB
   - 55 µL RNase-free water
   - 20 µL Proteinase K
2. Add 100 µL of the master mix to each sample.
3. Mix gently and briefly centrifuge.
4. Incubate at 56°C for 2 hours in a thermomixer without shaking.
5. Incubate samples at 90°C for 1 hour.
6. Briefly centrifuge and set the thermomixer to 65°C.
7. Remove and discard the upper blue phase while retaining the lower aqueous lysate.

##

## **RNA Removal and Protein Digestion**

1. Add 2 µL RNaseA to each sample.
2. Vortex and incubate at room temperature for 2 minutes.
3. Briefly, centrifuge.
4. Add 20 µL Proteinase K.
5. Vortex and incubate at 65°C for 15 minutes without shaking.
6. Briefly, centrifuge.

##

## **DNA Binding and Column Washing**

1. Add 250 µL Buffer AL and 250 µL ethanol to each sample.
2. Vortex thoroughly and briefly centrifuge.
3. Transfer 450 ul of the lysate to the QIAamp UCP MinElute column, centrifuge at 15000 x g for 30s
4. Transfer the residual lysate to the QIAamp UCP MinElute column, centrifuge at 15000 x g for 1 min, then discard the flow-through and reuse the collection tube
5. Add 500 ul buffer AW1 to each of the QIAamp UCP MinElute column, centrifuge at 15000 x g for 30s, then discard the flow-through and reuse the collection tube
6. Add 500 ul buffer AW2 to each of the QIAamp UCP MinElute column, centrifuge at 15000 x g for 30s, then discard the flow-through and reuse the collection tube
7. Add 250 ethanol to each spin column, centrifuge at 15000 x g for 30s
   1. Discard the flow-through and the collection tube.
   2. Place the spin column into a new collection tube and centrifuge for 3 min at full speed.

## **DNA Elution**

1. Transfer the column to a clean 1.5 mL microcentrifuge tube.
2. Add 20 µL Buffer ATE directly to the center of the membrane.
3. Incubate for 5 minutes at room temperature.
4. Centrifuge at maximum speed for 1 minute to elute DNA.
5. Store DNA at −20°C until further use.

## **Post-Extraction Quality Control**

- The following parameters must be recorded for every sample after DNA extraction

| **QC parameter** | **Method** | **Acceptance / typical value** |
| --- | --- | --- |
| DNA concentration and total yield | Fluorometric quantification (Qubit dsDNA HS assay) | Report ng/µL and total ng |
| Purity (protein) | Spectrophotometry (NanoDrop), **A260/A280** | ~1.8–2.0 (A ratio below ~1.8 suggests residual protein or organic carry-over; a ratio above ~2.0 suggests residual RNA) |
| Purity (salt / organic carry-over) | Spectrophotometry (NanoDrop), **A260/A230** | ≥ 1.8; flag < 1.5 (possible deparaffinization-solvent or salt carry-over) |
| DNA integrity | Agarose gel electrophoresis | FFPE DNA is expectedly fragmented. Excessive smearing indicates severe fragmentation and thus precludes use |

Because FFPE extracts are often low yield, the ratios obtained by NanoDrop can be unreliable at low concentration, so they must be interpreted together with the Qubit concentration and successful PCR amplification rather than as stand-alone pass/fail criteria.

- 1. **DNA Amplification**

1. Set up the PCR reactions using the LongAmp Taq 2X Master Mix (New England Biolabs, UK), which includes Taq DNA polymerase, dNTPs, MgCl_2_, and buffer components suitable for long-range amplifications.
2. Add specific primers targeting various regions of the 16S rRNA gene (~700-300 bp) to ensure broad microbial coverage.

### **Reaction mixture (per 25 µL reaction)**

| **Component** | **Volume (µL)** |
| --- | --- |
| LongAmp Taq 2X Master Mix | 12.5 |
| V3–V4 primers (0.7 forward + 0.7 reverse – from 10uM stock) | 1.4 |
| V4 primers (0.7 forward + 0.7 reverse – from 10uM stock) | 1.4 |
| V4–V5 primers (0.7 forward + 0.7 reverse – from 10uM stock) | 1.4 |
| V5–V7 primers (0.7 forward + 0.7 reverse – from 10uM stock) | 1.4 |
| Nuclease-free water | 4.4 |
| Template DNA | 2.5 |
| **Total volume** | **25.0** |

### Primers were described by Albers et al., 2023

1. Apply the following PCR cycling conditions:

### **PCR Cycling conditions**

| **Step** | **Temp** | **Time** | **Cycles** |
| --- | --- | --- | --- |
| Initial denaturation | 94 °C | 1 min | 1 |
| Denaturation | 94 °C | 20 s | 30 |
| Annealing | 59 °C | 30 s | 30 |
| Extension | 65 °C | 30 s | 30 |
| Final extension | 65 °C | 5 min | 1 |
| Hold | 4 °C | ∞ | — |

### **Post-PCR analysis**

- Run gel electrophoresis to confirm amplification and check for contamination.
- Include positive and negative controls in every PCR run to ensure accuracy and reliability.
- Successful amplification is confirmed for each sample by the presence of a clear band of the expected size on agarose gel electrophoresis before the product is carried forward to library preparation.
  1. **PCR Product Purification**

DNA purification was done using MinElute PCR Purification Kit (Qiagen, Germany), as follows:

1. Add 5 volumes of Buffer PB to 1 volume of PCR reaction and mix.
2. Place a QIAquick column in a 2 mL collection tube.
3. Apply the sample to the column and centrifuge for 60 s, then discard the flow-through and return the column to the same tube.
4. Add 750 µL Buffer PE and centrifuge for 60 s, then discard the flow-through and return the column to the same tube.
5. Centrifuge the empty column for 1 min to remove residual wash buffer.
6. Place the column in a clean 1.5 mL microcentrifuge tube.
7. Add 20 µL Buffer EB (10 mM Tris-Cl, pH 8.5) to the centre of the membrane, incubate for 5 min, then centrifuge for 1 min to elute.

**NOTE: Difference between Protocol 1 (P1) and Protocol 2 (P2)**

In Protocol 1 (P1), bacterial genomic DNA was selectively amplified using PCR (step 4.2), followed by purification of the PCR products (step 4.3) to remove non-target DNA, including a substantial proportion of host DNA.

FFPE-derived DNA is often highly fragmented; thus, residual host DNA fragments may still persist after a single amplification and purification step. Therefore, Protocol 2 (P2) incorporated an additional round of PCR amplification (step 4.2), and purification (step 4.3) to further enrich bacterial DNA and reduce the contribution of host DNA.

- 1. **Nanopore Library Preparation and Sequencing**

| **Parameter** | **Description** |
| --- | --- |
| Sequencer | Oxford Nanopore MinION Mk1C |
| Library kit | Native Barcoding Ligation Sequencing gDNA kit 96 V14 (SQK-NBD114.96) |
| Flow cell | R10 (FLO-MIN114) |
| Run duration | 48 h |

**Step 1: DNA Repair and End-Prep**

1. Combine per sample: 11 µL DNA sample, 1 µL diluted DNA Control Sample (DCS), 0.875 µL NEBNext FFPE DNA Repair Buffer, 0.875 µL Ultra II End-prep Reaction Buffer, 0.75 µL Ultra II End-prep Enzyme Mix, 0.5 µL NEBNext FFPE DNA Repair Mix.
2. Mix thoroughly by pipetting and spin down.
3. In a thermal cycler, incubate at 20 °C for 10 min, then 65 °C for 10 min.
4. Transfer each sample to a clean 1.5 mL DNA LoBind tube.
5. Resuspend AMPure XP beads (AXP) by vortexing.
6. Add 15 µL resuspended AXP to each reaction and mix by flicking. Incubate on a Hula mixer for 5 min at RT.
7. Prepare fresh 80% ethanol in nuclease-free water (allow ~400 µL per sample, plus excess).
8. Spin down and pellet the beads on a magnet until the eluate is clear; keep on the magnet and pipette off the supernatant.
9. Keeping the tube on the magnet, wash the beads with 200 µL fresh 80% ethanol without disturbing the pellet; remove and discard the ethanol.
10. Repeat the previous wash step.
11. Briefly spin, return to the magnet, and remove residual ethanol. Air-dry ~30 s (do not over-dry to cracking).
12. Remove from the magnet and resuspend the pellet in 10 µL nuclease-free water; spin down and incubate 2 min at RT.
13. Pellet the beads on the magnet until the eluate is clear.
14. Remove and retain 10 µL eluate into a clean 1.5 mL DNA LoBind tube. Quantify 1 µL using a Qubit fluorometer.
15. Take forward an equimolar mass of samples to be barcoded and pooled forward into the native barcode ligation step.

**Step 2: Native Barcode Ligation**

1. Thaw the Native Barcodes required for the number of samples at RT. Individually mix the barcodes by pipetting, spin down, and place them on ice.
2. Select a unique barcode for each sample to be run together on the same flow cell.
3. In 0.2 mL PCR tubes, add in order: 10 µL Blunt/TA Ligase Master Mix, 2.5 µL Native Barcode (NB01–24), 7.5 µL end-prepped DNA.
4. Mix gently by pipetting and spin down.
5. Incubate for 30 min at RT.
6. Pool the barcoded reactions, then add AMPure XP beads (AXP) for a 0.4X clean-up and mix by pipetting.
7. Incubate on a Hula mixer for 10 min at RT.
8. Prepare 2 mL fresh 80% ethanol in nuclease-free water.
9. Spin down and pellet on a magnet for 5 min until the eluate is clear; pipette off the supernatant.
10. Keeping on the magnet, wash with 700 µL fresh 80% ethanol without disturbing the pellet; remove and discard.
11. Repeat the previous wash step.
12. Spin down, return to the magnet, and remove residual ethanol. Air-dry ~30 s (do not over-dry).
13. Remove from the magnet and resuspend in 10 µL nuclease-free water by gentle flicking.
14. Pellet the beads on the magnet until the eluate is clear.
15. Remove and retain 10 µL eluate into a clean 1.5 mL DNA LoBind tube. Quantify 1 µL using a Qubit fluorometer.
16. Take forward the barcoded DNA library to the adapter ligation and clean-up step.

**Step 3: Adapter Ligation and Clean-Up**

1. In a 1.5 mL LoBind tube, mix in order: 30 µL pooled barcoded sample, 5 µL Native Adapter (NA), 10 µL NEBNext Quick Ligation Reaction Buffer (5X), 5 µL Quick T4 DNA Ligase.
2. Mix gently by pipetting and spin down.
3. Incubate for 30 min at RT.
4. Resuspend the AMPure XP beads (AXP) by vortexing.
5. Add 20 µL resuspended AXP to the reaction and mix by pipetting.
6. Incubate on a Hula mixer for 10 min at RT.
7. Spin down and pellet on the magnet; pipette off the supernatant.
8. Wash with 125 µL Short Fragment Buffer (SFB). Do not use ethanol — it is detrimental to the sequencing reaction. Flick to resuspend, spin down, return to the magnet, and remove the supernatant.
9. Repeat the previous wash step.
10. Spin down, return to the magnet, and remove residual supernatant.
11. Remove from the magnet and resuspend the pellet in 15 µL Elution Buffer (EB).
12. Spin down and incubate for 10 min at 37 °C; every 2 min, gently flick for ~10 s to encourage elution.
13. Pellet the beads on the magnet for ≥1 min until the eluate is clear.
14. Remove and retain 15 µL eluate (the DNA library) into a clean 1.5 mL DNA LoBind tube. Quantify 1 µL using a Qubit fluorometer.
15. Make up the library to 12 µL at 10–20 fmol. Store on ice until ready to load.

**Step 4: Priming and Loading the Flow Cell for Sequencing**

1. Prepare the priming mix by adding to the Flow Cell Flush (FCF) tube and mixing at RT: 1170 µL FCF, 30 µL Flow Cell Tether (FCT).
2. Open the device lid, slide the flow cell under the clip, and press down firmly to ensure good thermal/electrical contact.
3. Slide the priming-port cover clockwise to open the priming port.
4. Remove any air bubble: set a P1000 to 200 µL, insert into the priming port, and turn the wheel until the dial reads 220–230 µL (drawing back 20–30 µL). Never draw back more than 20–30 µL and keep the pore array covered by buffer at all times
5. Load 800 µL of the priming mix into the flow cell via the priming port, avoiding air bubbles. Wait 5 minutes; meanwhile prepare the library (next steps).
6. Mix the Library Beads (LIB) thoroughly by pipetting immediately before use
7. In a new 1.5 mL LoBind tube, prepare the library: 37.5 µL Sequencing Buffer (SB), 25.5 µL Library Beads (LIB) (or Library Solution, LIS), and 12 µL DNA library.
8. Gently lift the SpotON sample-port cover to expose the port.
9. Load 200 µL of the priming mix into the priming port (not the SpotON port), avoid air bubbles.
10. Mix the prepared library gently by pipetting just before loading.
11. Add 75 µL of the prepared library dropwise via the SpotON sample port, allowing each drop to flow in before adding the next.
12. Gently replace the SpotON cover (ensure the bung enters the port), close the priming port, and replace the device lid.
13. Begin the sequencing run and let it continue for up to 48 h.
14. **Expected sequencing Statistics**

Summary statistics for P1 and P2 protocols are given. They are dependent on the DNA input and quality of samples.

| **Metric** | **P1** | **P2** |
| --- | --- | --- |
| **Flow-cell / pore performance** | | |
| Initial available pores | ≈ 990 | ≈ 1,270 |
| Pore half-life (h) | ≈ 6 | ≈ 12 |
| **Sequencing output (MinKNOW run report)** | | |
| Total reads (million) | ≈ 1.6 | ≈ 4.1 |
| Total yield (Gb) | ≈ 0.85 | ≈ 2.55 |
| Read-length N50 (bp) | ≈ 415 | ≈ 520 |
| Basecalling pass rate (%) | ≈ 64 | ≈ 72 |

**References:**

Albers, Anne, Dorothee Cäcilia Spille, Eric Suero‐Molina, Frieder Schaumburg, Walter Stummer, Werner Paulus, and Christian Thomas. 2023. “Rapid Bacterial Identification from Formalin‐fixed Paraffin‐embedded Neuropathology Specimens Using 16S rDNA Nanopore Sequencing.” *Neuropathology and Applied Neurobiology* 49(1): e12871. doi:10.1111/nan.12871.

Jain, Miten, Hugh E. Olsen, Benedict Paten, and Mark Akeson. 2016. “The Oxford Nanopore MinION: Delivery of Nanopore Sequencing to the Genomics Community.” *Genome Biology* 17(1): 239. doi:10.1186/s13059-016-1103-0.
